# Helix-A peptide prevents gp120-mediated neuronal loss

**DOI:** 10.1186/s13041-019-0482-z

**Published:** 2019-06-25

**Authors:** Valeria Avdoshina, Francesca Taraballi, Ennio Tasciotti, Aykut Üren, Italo Mocchetti

**Affiliations:** 1 Department of Neuroscience, Laboratory of Preclinical Neurobiology, Georgetown University, EP09, New Research Building, 3970 Reservoir Rd., NW, Washington, DC 20057 USA; 2 Department of Regenerative Medicine, Houston Methodist Research Institute, Houston, TX USA; 3 Department of Oncology, Georgetown University Medical Center, Washington, DC USA; 4 Orthopedics and Sports Medicine, Houston Methodist Hospital, Houston, TX USA

**Keywords:** Dendritic simplification, HAND, HIV, Mesoporous nanoparticles, Microtubules, Neuronal survival

## Abstract

**Aim:**

The human-immunodeficiency virus (HIV) envelope protein gp120 promotes synaptic damage similar to that observed in people living with HIV who have neurocognitive disorders. The neurotoxic effect of gp120 appears to occur through the α-helix motif that binds to neuronal microtubules (MTs). In this study, we examined the ability of short peptide derivatives from Helix-A, a peptide synthesized based on α-helix structure of gp120, to displace gp120 from binding to MTs and prevent its neurotoxic effects.

**Methods:**

Surface plasmon resonance was used to determine the binding of Helix-A and its modifications to MTs. Helix-A peptide and derivatives were delivered inside rat primary cortical neurons by mesoporous silica nanoparticles (MSN). Neuronal processes and survival were evaluated by microtubule associated protein 2-immunostaining and Hoechst/Propidium iodide, respectively.

**Results:**

Surface plasmon resonance analysis revealed that Helix-A but not its modifications binds to MTs. Also, only Helix-A MSN but not other peptides prevented the ability of gp120 to reduce neuronal processes as well as neuronal survival. Thus, the amino acid structure of Helix-A is key for its neuroprotective activity.

## Main text

Various degrees of axonal and dendritic pathology are seen in human-immunodeficiency virus (HIV) positive subjects who develop HIV-associated neurocognitive disorders (HAND) [1, 2]. This neurological consequence of HIV infection of the brain occurs also in the HIV-infected population undergoing the antiretroviral therapy [3]. Thus, new therapeutic strategies to reduce neuronal loss are needed. However, more knowledge about the mechanisms of how HIV is neurotoxic is crucial for a better adjunct therapy.

HIV proteins are, among other causes, most likely responsible for the damage of synapses. In particular, the envelope protein gp120, which can be secreted by HIV-infected cells, induces neuronal apoptosis through direct binding to chemokine receptors, CXCR4 and CCR5 [4, 5]. Binding of gp120 to these receptors promotes its rapid endocytosis by neurons [6]. Importantly, internalized gp120 binds to microtubules (MTs) [7, 8], which are the major avenue for intracellular transport of many organelles, including mitochondria, and synaptic vesicles to distal axons and dendrites. Changes in the integrity of MTs are sufficient to alter proper energy supply within neurites and synapses leading to acute or chronic axonal and dendritic fragmentation [9]. Thus, gp120 by binding to MTs may disrupting their function and decrease neuronal survival.

We have previously provided evidence that the conserved α-helix region of gp120 binds to the carboxy terminal tail of class III beta tubulin (TUBB3), one of the MT elements found predominantly in neurons [8]. When such binding is blocked by a 19-amino acid peptide, termed Helix-A, synthetized based on the α-helix motif of gp120, the neurotoxic effect of the envelope protein is abolished [8]. In this work, we have analyzed different peptides carrying various amino acid mutations to investigate whether the α-helix motif is necessary for the neuroprotective activity of Helix-A.

**Fig. 1:**
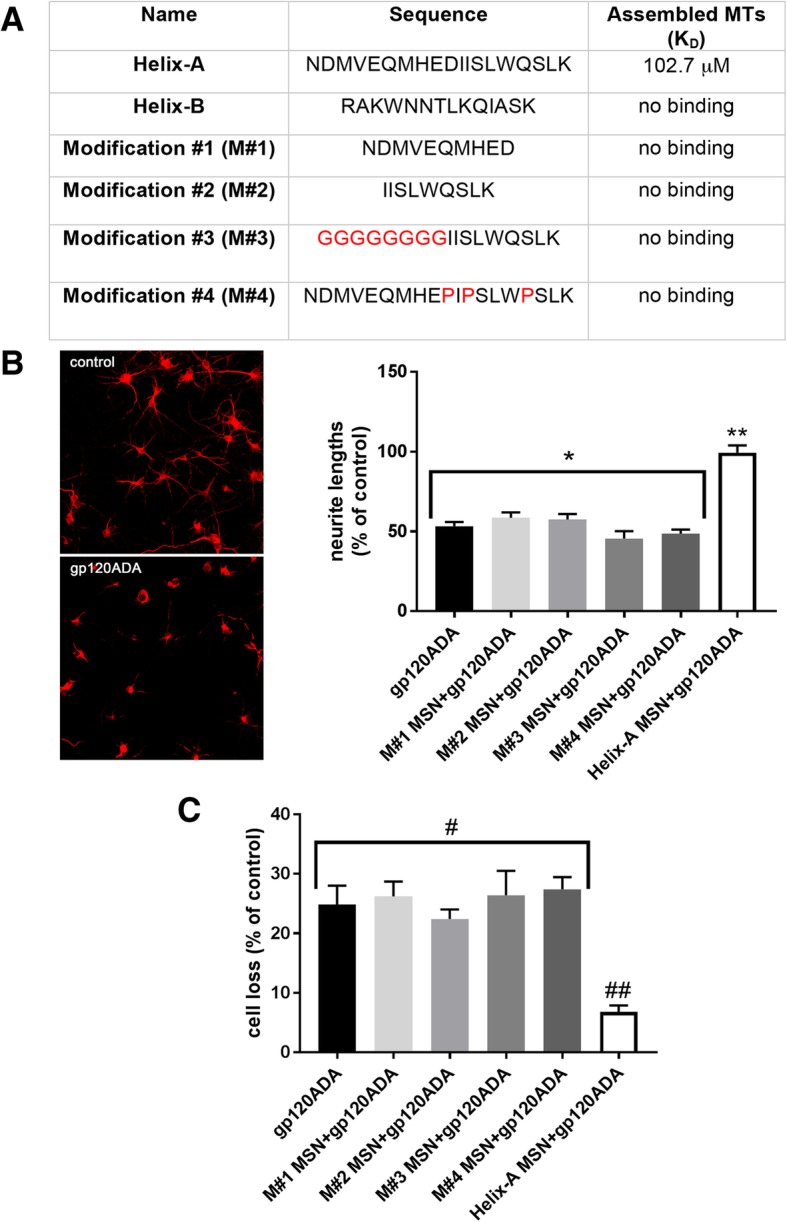
The neuroprotective effect of peptides against gp120 depends on their binding to MTs. **a**. Helix-A and other peptides were purchased from Genscript, Piscataway, NJ. The binding affinity of peptides to assembled MTs (Cytoskeleton Inc., Denver, CO), expressed as K_D_, was determined by surface plasmon resonance (Biacore™ T200, GE Health care Bio-Science, Piscataway, NJ) as previously described [8]. **b-c**. Rat cortical cultures were prepared from embryonic day 14–15 Sprague Dawley rats (Taconic, Derwood, MD) as previously described [8]. Neurons were exposed to gp120ADA (5 nM) (Immunodiagnostics Inc., Woburn, MA) alone or in combination with Helix-A MSN or the other four modified peptides crosslinked to MSNs (5 μM each), for 24 h. Neurite length (**a**) and cell survival (**b**) were then determined as previously described [8]. Boiled gp120ADA was used as a control. **b**. Example of images of neurons (20×) stained with a mouse MAP2 antibody (1:5000; MilliporeSigma, St Louis, MO) after exposure to boiled gp120 (control) or gp120ADA for 24 h. Neurite lengths were determined by analyzing MAP2 immunoreactivity in three randomly selected fields (10 neurons per field) from three separate experiments using ImageJ, as detailed elsewere [8]. *p < 0.0001 vs control, **p < 0.0001 vs gp120; One-way ANOVA followed by multiple comparisons by Tukey’s test. **c**. Neuronal survival was determined by Hoechst/PI staining (MilliporeSigma) as previously described [8]. Results are expressed as mean ± SEM (from 3 separate experiments with 2 duplicates per condition). #*p* < 0.005 vs control, ##*p* < 0.0004 vs gp120; One-way ANOVA followed by multiple comparisons by Tukey’s test

To examine the structure of Helix-A required to displace gp120 from binding to TUBB3, we designed and synthetized peptides to carry mutations so that either the helix motif of Helix-A is lost or specific amino acid sequences are changed (Fig. 1a). These include peptides with a truncated C-terminal (NDMVEQMHED, modification #1) or N-terminal (IISLWDQSLK, modification #2) of Helix-A. These modifications produce peptides with shorter helical structure. In addition, we synthetized peptides in which glycine (modification #3) or proline (modification #4) were introduced into the original Helix-A peptide to completely distrupt the α-helix conformation. We also synthetized Helix-B peptide, which has a α-helix structure but it does not displace gp120 from MTs [8]. Analysis of interaction of peptides to assembled MTs, using surface plasmon resonance, indicated that only the unmodified Helix-A peptide binds to assembled MTs (Fig. 1a). Binding of Helix-A peptide to TUBB3 was in the micromole range; however, this is consistent with a rapid dissociation of Helix-A from MTs due to its small mass (~ 1696 Da). Nevertheless, our data support the suggestion that the amino-acid sequence as well as the α-helix structure of Helix-A peptide are essential for its binding to MTs.

We have previously shown that Helix-A peptide cannot cross cell membranes unless cross-linked to mesoporous silica nanoparticles (MSN) [10]. Once inside neurons, Helix-A MSN has neuroprotective effect against at least two strains of gp120: T-tropic (gp120IIIB) and M-tropic (gp120ADA) [8]. To examine whether Helix-A peptide modifications are neuroprotective, we cross-linked all modified peptides to MSN. We then used these peptides to test whether they prevent gp120ADA-mediated neurite pruning and cell loss in rat cortical cultures. MSN alone or peptides crosslinked to MSN alone did not affect either neurite lengths, determined by microtubule associated protein 2 (MAP2) staining, or neuronal survival measured by Hoechst/propidium iodide (PI) (data not shown), suggesting that these compounds have no side effects tha impair neuronal viability. However, Helix-A MSN but not all modified peptide-MSN prevented gp120-mediated neurite pruning (Fig. 1b), as well as neuronal loss (Fig. 1c). It is important to note that Helix-A not crosslinked to MSN or Helix-B MSN were ineffective in blocking gp120 neurotoxicity [8]. These data support the hypothesis that the amino acid sequence of the Helix-A, which binds to MTs, is crucial for the neuroprotective activity against gp120.

MTs are composed of tubulin heterodimers comprised of repeats of α- and β-tubulin, which are conserved among all eukaryotic species [11]. TUBB3 contains multiple α-helix motifs. The secondary structure of gp120 also contains three α-helix motifs that can determine associations between different proteins [12]. We have previously shown that the α-helix of gp120 near the V3 loop could form a dimer with the α-helix of TUBB3 located in its carboxyl terminal tail [13]. Such binding may alter the stability and function of neuronal MTs. Thus, a binding of gp120 to TUBB3 may impair MTs function. Indeed, we have previously demonstrated that the Helix-A peptide, which displaces gp120 binding to TUBB3 by interrupting the helix-helix interaction of gp120 with TUBB3, is neuroprotective [8]. In this work, we further established that modifications of the amino acid sequence in Helix-A peptide that either disrupt its helical conformation or abolish the binding site as well as the neuroprotective activity against gp120. Nevertheless, we also observed that another α-helix motif (Helix-B) in gp120 secondary structure does not bind to MTs and it is not neuroprotective. Therefore, both the α-helix structure, as well as the amino acid sequence within, are equally important for high affinity binding of gp120 to MTs. Lastly, the amino acid sequence of the α-helix structure is not homologous to that found in the V3 loop domain necessary for the binding of gp120 to the chemokine co-receptors [14]. Thus, our results support the suggestion that activation of these receptors, although necessary to deliver gp120 inside neurons, [10], may not entirely explain the cellular mechanisms of gp120 neurotoxicity.

The ability of Helix-A to block gp120 neurotoxicity was tested in an in vitro experimental model of HAND. Future studies must test the effect of Helix-A in vivo to ascertain whether this peptide is a suitable approach for a new adjunct therapy against HIV, in addition to CXCR4 or CCR5 receptor antagonists.

## Data Availability

All data generated or analyzed during this study are included in this published article.

## Abbreviations

HAND: HIV-associated neurocognitive disorders
HIV: human-immunodeficiency virus
MAP2: microtubule associated protein 2
MSN: mesoporous silica nanoparticles
MTs: microtubules
TUBB3: class III beta tubulin
